# Does Secondary Ischemia Time After Failed Digit Replantation Predict Survival?

**DOI:** 10.1016/j.jhsg.2026.101115

**Published:** 2026-08-07

**Authors:** Ole-Gunnar Olsen, Ida Neergård Sletten, Magne Røkkum, Johanne Korslund, Wender Figved, Rasmus Dehli Thorkildsen

**Affiliations:** ∗ Unit for Hand- and Microsurgery, Division of Orthopedic Surgery, Oslo University Hospital, Oslo, Norway; † Faculty of Medicine, University of Oslo, Oslo, Norway; ‡ Division of Orthopedic Surgery, Oslo University Hospital, Oslo, Norway; § Vestre Viken Hospital Trust, Division of Orthopedic Surgery, Gjettum, Norway

**Keywords:** Arterial insufficiency, Replantation, Secondary ischemia

## Abstract

**Purpose:**

Early identification of vascular compromise following digit replantation and revascularization, and prompt restoration of blood flow is considered critical for successful salvage. This study aimed to assess the duration of secondary ischemia in replanted and revascularized digits with postoperative arterial insufficiency that were reoperated on and to determine whether there is a correlation between secondary ischemia time and digit survival.

**Methods:**

We identified replanted and revascularized digits reoperated for isolated arterial thrombosis during their primary admission at the National Unit for Replantation Surgery in Norway from 2010 to 2024. Ischemia onset was defined as a decrease in surface skin temperature of 1 °C per hour or 1.5 °C over 2 hours. Data on the onset of ischemia, time to reestablishment of arterial circulation, and survival were extracted from the unit’s internal quality registry. Well-known predictors for survival following digit replantation were also assessed to control for confounding variables. We conducted binary logistic regression and compared the means of secondary ischemia time between the survival and nonsurvival groups with a *t* test.

**Results:**

A total of 59 digits with vascular compromise were reoperated for isolated arterial thrombosis. Logistic regression did not identify secondary ischemia time as a predictor for survival. The average secondary ischemia time for salvaged digits compared with nonsalvaged digits was not different. The survival and nonsurvival groups were similar regarding confounding variables.

**Conclusions:**

Digit replantations that undergo postoperative ischemia due to arterial occlusion may tolerate longer secondary ischemia times than previously assumed. Our study did not demonstrate secondary ischemia due to arterial thrombosis as a critical predictor for salvage. Evidence of a critical secondary ischemia threshold is still lacking.

**Clinical relevance:**

This study may support a more nuanced urgency in re-exploration decisions after digit replantations with postoperative arterial insufficiencies. Delayed recognition of arterial insufficiency can still result in successful salvage.

Primary ischemia duration following digit amputation (time from injury to restoration of blood flow) is considered a predictive factor for survival after replantation. A primary ischemia time of up to 12 hours is cited as a tolerable limit.^1^ Harbour et al^2^ showed that there is little evidence supporting primary ischemia time thresholds, and there is growing evidence supporting equal survival rates for delayed replantation.^3^^,^^4^ Successful digit replantations have been reported after primary ischemia times up to 94 hours.^5^

Close monitoring of blood circulation after replantation is integral to most replantation protocols, as early identification of circulatory insufficiency and prompt restoration of blood flow are considered crucial to prevent irreversible tissue damage. To our knowledge, no evidence regarding the importance of secondary ischemia time on survival rates. Some have postulated that critical secondary ischemia times may be half that of primary ischemia times, suggesting minimal chances of digit salvage after 4−6 hours.^6^

Experimental studies on free flaps in animal models indicate that the secondary critical ischemia times are significantly shorter than those for primary ischemia, with venous occlusion resulting in worse outcomes than arterial occlusion.^7^, ^8^, ^9^ Kerrigan et al^10^ reported an average secondary critical ischemia time (50% flap loss) of 7.2 hours in experimental skin flaps. In comparison, the same author showed that the average primary critical ischemia time was 13 hours.

Although published survival rates after digit replantation range from 53% to 96%, the reported salvage rates following circulatory impairment, arterial/venous etiology combined, vary from 12% to 44%. Circulatory impairment due to venous congestion may have a poorer prognosis compared with arterial occlusion.^11^, ^12^, ^13^, ^14^, ^15^

The main objective of this study was to examine whether secondary ischemia time due to isolated arterial occlusion predicts survival following reoperation for failed digit replantation. Secondary objectives were to analyze other risk factors for digit survival after secondary ischemia and compare survival and nonsurvival groups. We hypothesized that secondary ischemia time is a predictor of digit salvage.

## Materials and Methods

In this retrospective cohort study, the internal quality registry, approved by the hospital’s Data Protection Officer, identified all digit replantations and revascularizations from 2010 to 2024 at the Oslo University Hospital, the National unit for replantation in Norway. Information was collected from the patients’ medical records. The study was conducted in accordance with the Strengthening the Reporting of Observational Studies in Epidemiology guidelines.^16^

**Figure 1:**
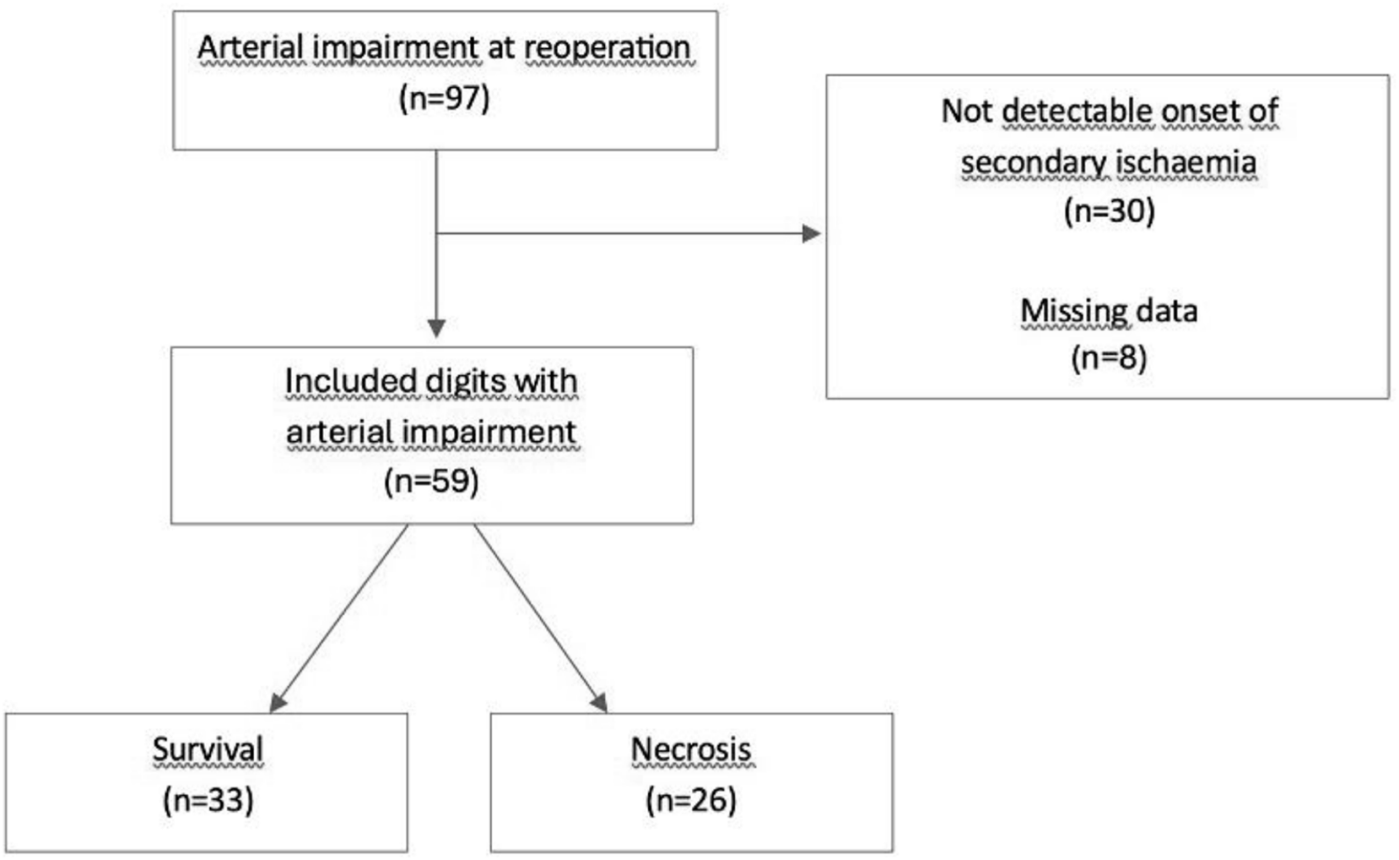
Flow chart of study inclusion. Flow diagram showing identification, inclusion, and exclusion of replanted and revascularized digits reoperated for secondary ischemia due to isolated arterial occlusion during the study period.

The term “replantation” designates the reattachment and reestablishment of blood flow of a completely severed body part, although “revascularization,” in this material, was used whenever the injured and circulatory-deprived body part had a skin connection to the rest of the body, and venous drainage was presumed intact. We included all replanted and revascularized digits distal to the distal metacarpal metaphysis (Tamai zone V) with secondary ischemia due to isolated arterial occlusion confirmed at reoperation. Cases in which the onset of ischemia could not be established or with missing data were excluded (Fig. 1).

The monitoring protocol involved hourly assessments of skin temperature, color, and capillary refill for 10 days. Ischemia was defined as a drop in the hourly temperature registration of 1 °C/h or 1.5 °C/2 hours. The onset was set to the middle of the relevant time interval. The time of restoration of blood flow during the reoperation was registered. For the analyses, the duration of the secondary ischemia was rounded to the nearest 15 minutes. The time when it was decided to reoperate was noted to evaluate the sensitivity of identifying arterial impairment using standard monitoring techniques.

Six hundred forty-three patients underwent replantation/revascularization during the study period. Fifty-nine digits (9%) in 54 patients (48 men and 6 women) with a mean age of 47.8 years (95% confidence interval [CI], 42.6−52.9) met the criteria and were included in the study (Fig. 1).

In 29 digits, restoration of arterial inflow was sufficient. The remaining 30 digits required additional venous anastomoses. In 52 digits, one artery was repaired. Two arteries were repaired in seven digits. Five patients underwent reoperation for arterial insufficiency in two fingers. For two of the patients, revascularizations of the two fingers were undertaken in separate surgeries (ie, on different days), whereas in the remaining three patients, the two digits were revascularized during the same procedure. In these cases, surgery was planned after circulatory failure was suspected in one finger. In the preoperative wait for theater availability, ongoing monitoring indicated circulatory failure in finger 2, giving two separate timepoints for the onset of ischemia. All fingers were monitored individually, and separate arteries were repaired both during the initial procedure and at reoperation.

At reoperation, arterial inflow was restored with direct suture in 13 digits and vein graft in 46 digits. Vein grafts were harvested from the volar aspect of the ipsilateral forearm. One artery was repaired in 53 digits, two arteries in six digits. After arterial flow was reestablished at reoperation, intact venous drainage was confirmed by direct visualization of the prior venous anastomosis in 17 cases, otherwise by clinical inspection of the digit. As during the primary procedure, heparin (2,500 IU) was administered upon release of the vascular clamps following arterial repair. Anticoagulation protocol (delteparin 5,000 IU and acetylsalicylic acid 75 mg daily) was continued according to the hospital routine.

Continuous variables are presented as means with 95% CIs. Categorical variables are presented in numbers. Due to the lack of prior justified estimates of a critical secondary ischemia time after replantation and estimates of effect sizes, we did not perform an a priori power analysis.

The dependent variable in the logistic regression model of factors associated with digit survival was the absence of revision amputation due to necrosis within 3 months following the initial surgery. The main independent variable was secondary ischemia time, and secondary variables were patient age, diabetes/cardiovascular disease, smoking habit, postoperative day of circulatory impairment, mechanism of trauma (guillotine, saw, log splitter, crush, avulsion), thumb replantation, level of injury (Tamai classification), vein grafting at reoperation, and type of surgical procedure (replantation vs revascularization).

**Figure 2:**
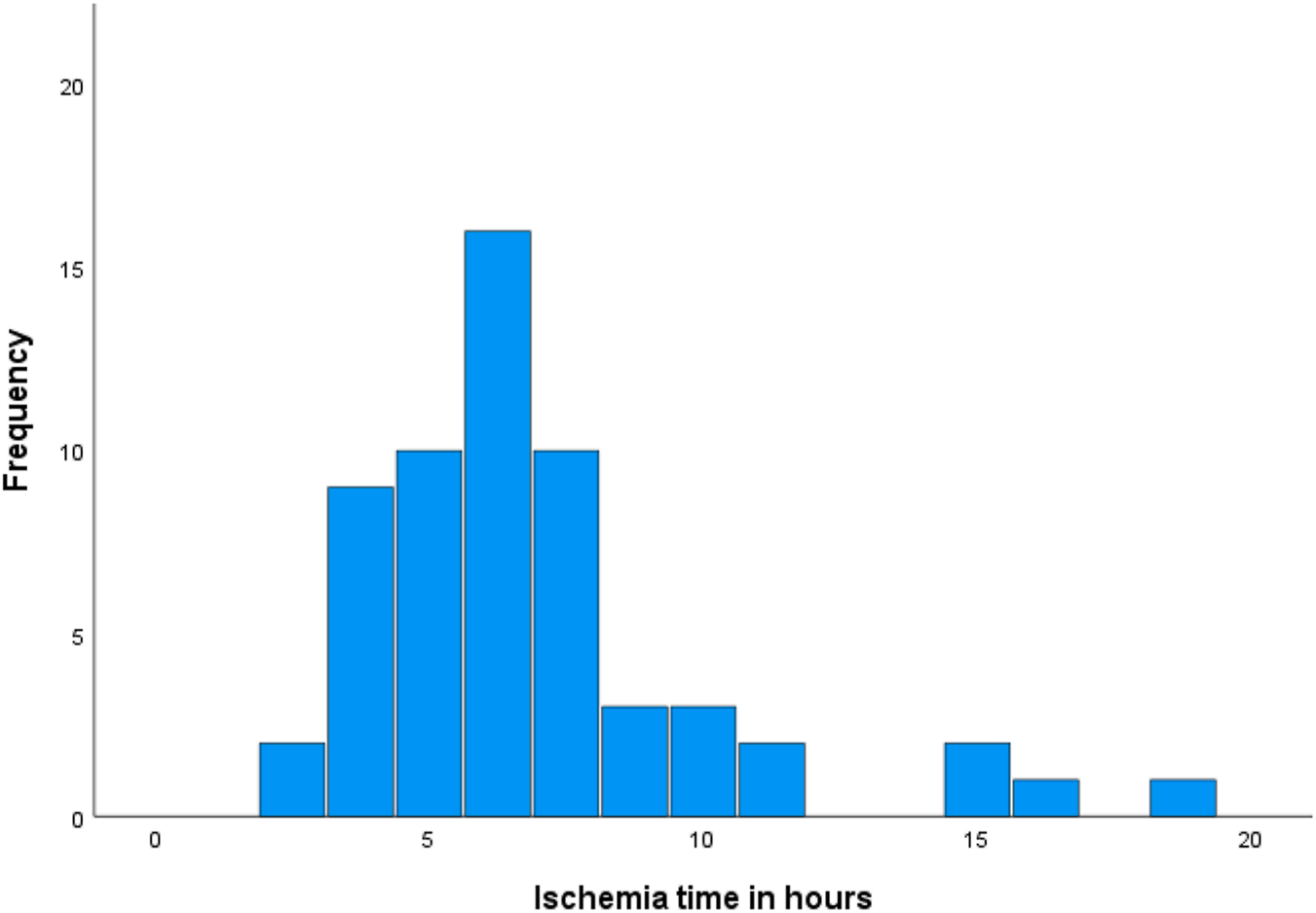
Distribution of secondary ischemia times. Histogram showing the distribution of secondary ischemia duration (in hours) among digits reoperated for isolated arterial thrombosis.

Univariate and multivariate binary logistic regression were applied to analyze predictors for survival, as well as an independent *t* test across salvage and nonsalvage groups. In the multivariate analysis, we decided to adjust for the patient-specific variables: age, diabetes/cardiovascular disease, and smoking habits. Significance level was set to .05. We treated each digit as an independent statistical unit. In the regression analysis, one time unit was set to 15 minutes. Normality of the main independent variable was assessed by visual inspection of the histogram and Q-Q plot (Fig. 2).

## Results

The overall salvage rate after reoperation for secondary ischemia was 56% (n = 33). The mean secondary ischemia time was 6.9 hours (95% CI, 6.0−7.7). One digit was salvaged 15.5 hours after the onset of secondary ischemia. The decision to proceed with reoperation occurred a mean of 2.9 hours (95% CI, 2.1−3.7) after the onset of ischemia. The survival and nonsurvival groups were similar regarding all background variables (Table 1).

**Table 1:** Distribution of Variables in Salvage and Necrosis Groups

Variable	Salvage (n = 33)	Necrosis (n = 26)
Patient age in y (95% CI)	45 (38−51)	52 (44−60)
Diabetes mellitus/cardiovascular disease (n)	6	6
Smoking (n)	11	9
Mechanism of trauma (n)		
Saw	21	16
Log splitter	4	6
Crush	4	4
Avulsion	2	2
Tamai level of injury (n)		
3	17	8
4	12	18
5	4	0
Type of revascularization (n)		
Replantation (artery and vein)	17	13
Recirculation (artery only)	16	13
Graft at reoperation (n)	26	20
Thumb revascularization (n)	14	5
Postoperative day of secondary ischemia onset (n)		
0	10	12
1	12	7
2	4	1
3	3	4
4	3	2
5	0	0
6	1	0

In the regression model, we found that secondary ischemia time was not a predictor of digit survival. The adjusted odds ratio per one-unit increase in secondary ischemia time was .92 (95% CI, 0.77−1.08, *P* = .31), a nonsignificant 8% lower odds of survival per 15-minute increase in secondary ischemia time. Neither were any of the secondary variables associated with digit survival (Table 2). In the *t* test, the salvage group had a mean secondary ischemia time of 6.4 hours (95% CI, 5.5−7.3), compared with 7.4 hours (95% CI, 5.8−9.0) in the nonsalvage group (*P* = .25).

**Table 2:** Univariate and Multivariate Regression Analysis, Crude, and Adjusted Values

Variable	Nonadjusted OR (95% CI)	*P*	Adjusted OR (95% CI)	*P*
Secondary ischemia time	0.91 (0.77−1.07)	.25	0.92 (0.77−1.08)	.31
Age	0.98 (0.96−1.01)	.17	0.98 (0.95−1.01)	.23
Diabetes mellitus/cardiovascular disease	0.74 (0.21−2.64)	.64	1.20 (0.26−5.48)	.81
Smoking	0.92 (0.32−2.79)	.94	0.93 (0.31−2.84)	.90

OR, odds ratio.

## Discussion

In this study of 59 replanted and revascularized digits with secondary vascular compromise due to arterial thrombosis, secondary ischemia time was not found to be a predictor for survival.

The observed confidence interval for the mean secondary ischemia time may explain why we could not demonstrate an effect. As Kerrigan et al^10^ demonstrated in free flaps, the relationship between secondary ischemia time and survival follows a sigmoid curve. Up to a certain point, secondary ischemia time is well tolerated, whereafter the negative effect on survival becomes evident. Our results indicate that the critical secondary ischemia time after replanted digits that fail because of arterial thrombosis is beyond the previously proposed 4−6 hours and likely beyond 7 hours, as demonstrated in experimental free flaps.^1^^,^^7^ Similar to the growing evidence of equal survival rates after delayed digit replantations, secondary ischemia due to arterial occlusion may be better tolerated than previously expected.

This study focused solely on secondary ischemia because of arterial impairment at the digit level, a condition seemingly well tolerated. More proximal amputations involve vulnerable muscle tissue. We excluded venous and combined circulatory failures, as identifying the onset of ischemia proved more challenging. Animal studies on free flaps suggest venous congestion results in greater tissue toxicity with shorter tolerable secondary ischemia time.^7^^,^^8^ This corresponds to our clinical experience with venous congestion in replanted digits.

Waikakul et al^17^ found that prolonged primary ischemia was not a major factor for survival, but it affected the functional outcome, assessed with Chen et al^5^ classification. Two-point discrimination was significantly poorer in digits with longer primary ischemia time than 9 hours. Secondary ischemia may not differ in this aspect, but this was beyond the scope of the present project.

To our knowledge, there is no prior study investigating the effect of secondary ischemia time on salvage after replantation. The strengths of our study are that we investigated all replantations and revascularizations during a 15-year period at a national center, ensuring capture of all patients/injuries in the country. We based our analysis on predetermined measurements for the onset of ischemia and the timing of recirculation.

We cannot conclude that secondary ischemia time is irrelevant as a predictor for salvage. The retrospective design and the small sample size are important limitations of the study. In 30 digits affected by arterial thrombosis, we were unable to determine the onset of ischemia based on our predefined criteria. Most of these cases did not recover after the primary surgery, raising the question whether they were actually, or more than briefly, revascularized during the primary procedure. Although this may limit the generalizability of our findings, we consider this group to represent a different clinical entity.

It is not possible to define the exact time of ischemia onset in the clinical setting with intermittent clinical examination. Our predefined criteria for ischemia onset based on a given temperature drop may be somewhat arbitrary. In our unit, this temperature reduction has traditionally acted as a “red flag” indicating potential vascular compromise. Clinical observations of skin color and capillary refill as indicators of ischemia onset were not factored into the analysis because of their subjective nature.

We decided to include cases where more than one finger was revascularized during reoperation. This reflects the everyday setting in which single fingers, apart from the thumb, are seldom candidates for replantation. We recognize that the assumption of independence between fingers may be questioned. Although all included fingers received their arterial input via separate digital arteries (ie, no proximal anastomoses on the common palmar digital arteries), systemic factors such as a fall in blood pressure or local factors in the hand may have affected more than one digital artery. The three patients who required reoperation of two digits during the same surgery may be examples of this.

Future research should include larger patient cohorts or address the impact of secondary ischemia time resulting from venous or combined insufficiencies. The latter is more challenging, as the onset of ischemia due to venous insufficiency is more difficult to determine. More precise monitoring methods may be a prerequisite for conducting such studies.

## Conflicts of Interest

No benefits in any form have been received or will be received related directly to this article.

## References

[1] Wolfe V.M., Wang A.A. (2015). Replantation of the upper extremity: current concepts. J Am Acad Orthop Surg.

[2] Harbour P.W., Malphrus E., Zimmerman R.M., Giladi A.M. (2021). Delayed digit replantation: what is the evidence?. J Hand Surg Am.

[3] Cavadas P.C., Rubí C., Thione A., Pérez-Espadero A. (2018). Immediate versus overnight-delayed digital replantation: comparative retrospective cohort study of survival outcomes. J Hand Surg Am.

[4] Lin I.C.F., Yoon A.P., Kong L., Wang L., Chung K.C. (2022). Association between daytime vs overnight digit replantation and surgical outcomes. JAMA Netw Open.

[5] Wei F.C., Chang Y.L., Chen H.C., Chuang C.C. (1988). Three successful digital replantations in a patient after 84, 86, and 94 hours of cold ischemia time. Plast Reconstr Surg.

[6] Wolfe S.W., Pederson W.C., Kozin S.H., Cohen M.S. (2016). Green’s operative hand surgery.

[7] Kerrigan C.L., Zelt R.G., Daniel R.K. (1984). Secondary critical ischemia time of experimental skin flaps. Plast Reconstr Surg.

[8] Hauge E.M., Balling E., Hartmund T., Hjortdal V.E. (1997). Secondary ischemia caused by venous or arterial occlusion shows differential effects on myocutaneous island flap survival and muscle ATP levels. Plast Reconstr Surg.

[9] Kerrigan C.L., Wizman P., Hjortdal V.E., Sampalis J. (1994). Global flap ischemia: a comparison of arterial versus venous etiology. Plast Reconstr Surg.

[10] Kerrigan C.L., Daniel R.K. (1982). Critical ischemia time and the failing skin flap. Plast Reconstr Surg.

[11] Ma Z., Guo F., Qi J., Xiang W., Zhang J. (2016). Effects of non-surgical factors on digital replantation survival rate: a meta-analysis. J Hand Surg Eur Vol.

[12] Sears E.D., Chung K.C. (2011). Replantation of finger avulsion injuries: a systematic review of survival and functional outcomes. J Hand Surg Am.

[13] Hatchell A.C., Sandre A.R., McRae M., Farrokhyar F., Avram R. (2019). The success of salvage procedures for failing digital replants: a retrospective cohort study. Microsurgery.

[14] Elmaraghi S., Israel J.S., Gander B. (2022). Systematic review of replant salvage and cost utility analysis of inpatient monitoring after digit replantation. J Hand Surg Am.

[15] Morrison W.A., O’Brien B.M., MacLeod A.M. (1977). A long-term review of digital replantation. Aust N Z J Surg.

[16] von Elm E., Altman D.G., Egger M. (2008). The Strengthening the Reporting of Observational Studies in Epidemiology (STROBE) statement: guidelines for reporting observational studies. J Clin Epidemiol.

[17] Waikakul S., Sakkarnkosol S., Vanadurongwan V., Un-nanuntana A. (2000). Results of 1018 digital replantations in 552 patients. Injury.

